# Diagnosis of Salivary Gland Tumors Using Transfer Learning with Fine-Tuning and Gradual Unfreezing

**DOI:** 10.3390/diagnostics13213333

**Published:** 2023-10-29

**Authors:** Ping-Chia Cheng, Hui-Hua Kenny Chiang

**Affiliations:** 1 Department of Biomedical Engineering, National Yang Ming Chiao Tung University, Taipei 11221, Taiwan; i.cruising@gmail.com; 2 Department of Otolaryngology Head and Neck Surgery, Far Eastern Memorial Hospital, New Taipei City 22060, Taiwan; 3 Department of Communication Engineering, Asia Eastern University of Science and Technology, New Taipei City 22060, Taiwan

**Keywords:** salivary gland tumor, ultrasound, deep learning, convolutional neural network, transfer learning, gradient-weighted class activation mapping (Grad-CAM)

## Abstract

Ultrasound is the primary tool for evaluating salivary gland tumors (SGTs); however, tumor diagnosis currently relies on subjective features. This study aimed to establish an objective ultrasound diagnostic method using deep learning. We collected 446 benign and 223 malignant SGT ultrasound images in the training/validation set and 119 benign and 44 malignant SGT ultrasound images in the testing set. We trained convolutional neural network (CNN) models from scratch and employed transfer learning (TL) with fine-tuning and gradual unfreezing to classify malignant and benign SGTs. The diagnostic performances of these models were compared. By utilizing the pretrained ResNet50V2 with fine-tuning and gradual unfreezing, we achieved a 5-fold average validation accuracy of 0.920. The diagnostic performance on the testing set demonstrated an accuracy of 89.0%, a sensitivity of 81.8%, a specificity of 91.6%, a positive predictive value of 78.3%, and a negative predictive value of 93.2%. This performance surpasses that of other models in our study. The corresponding Grad-CAM visualizations were also presented to provide explanations for the diagnosis. This study presents an effective and objective ultrasound method for distinguishing between malignant and benign SGTs, which could assist in preoperative evaluation.

## 1. Introduction

**Figure 1:**
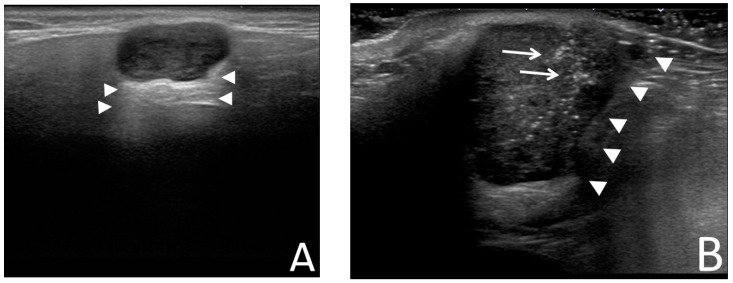
The subjective ultrasound features of SGTs. Note: (**A**) Benign SGTs are usually characterized by well-defined, homogeneous, and solid masses with posterior enhancement (arrowheads). (**B**) On the other hand, malignant SGTs tend to exhibit indistinct boundaries, irregular shapes (arrowheads), the presence of calcifications (arrows), and a lack of posterior enhancement. Abbreviation: SGT: salivary gland tumor.

Salivary gland tumors (SGTs) refer to the abnormal growth of cells within the salivary glands, which can present as swelling near the auricular region or below the jaw [1]. The incidence of SGTs ranges from 0.4 to 13.5 cases per 100,000 people [2,3,4]. According to the 5th Edition World Health Organization classification, there are 15 types of benign SGTs and 22 types of malignant SGTs [5]. However, diagnoses of SGTs can be challenging due to the heterogeneous histology and varying degrees of malignancy among different SGTs, even with the aid of imaging examinations and fine needle aspiration cytology (FNAC) [6,7]. The diagnosis of SGT is crucial for treatment planning. Benign tumors may only require monitoring or surgical removal, while malignant tumors typically necessitate more aggressive treatments [8]. The significance of noninvasive diagnostic tools has been highlighted in previous studies [9,10]. Among imaging examinations, ultrasound (US) remains the primary imaging tool for evaluating SGTs due to its affordability, lack of radiation exposure, and capability to perform FNAC simultaneously. However, diagnosing SGTs using US relies on subjective features (Figure 1) [11,12,13]. Different specialists may interpret the same images differently. Therefore, our aim was to establish objective methods that can assist in the diagnosis of SGTs using US images.

With advancements in computing power and GPUs, deep learning (DL) has become increasingly utilized in medical image analysis [14]. Convolutional neural networks (CNNs), which consist of convolutional layers, pooling layers, and fully connected layers, are widely used for medical image classification [15]. In CNNs, convolutional layers extract feature maps from input images using kernels, while pooling layers reduce the dimensions of the feature map by consolidating multiple pixels into a single value. These processes enable the selection of high-level features from the input image to aid in classification. However, training a deep CNN requires a large quantity of labeled data, which can be difficult to obtain, especially in fields such as medical image analysis, where expert annotation is needed [16]. Transfer learning (TL) represents using a model trained on one task and applying it to a different task [17]. In medical image analysis, TL often employs a pretrained model from a large dataset, such as ImageNet, and applies it to a new task with a small sample size, such as US image classification [18]. Although US images are grayscale, which differs from the color images in imageNet, the pretrained model still performs well in grayscale image classification [19]. One study utilized several TL models to evaluate SGTs using US images [20]. They included 176 SGTs in the training set and 75 SGTs in the validation set. The diagnostic accuracy for differentiating between malignant and benign SGTs was 79% for ResNet50, 77% for DenseNet121, 80% for EfficientNetB3, 81% for ViT-B\16, and 77% for experienced radiologists. However, these results are still not satisfactory.

Fine-tuning is a technique that involves freezing some of the bottom layers of a network and only training the top layers on new data [21]. Studies have demonstrated that TL with fine-tuning can achieve higher diagnostic performance than training a CNN from scratch [22]. Another technique is gradual unfreezing [23], which was initially introduced in the ULMFit model [24]. This technique gradually unfreezes layers from top to bottom during the training process. By doing so, the model can update its weights while retaining its previous knowledge when applied to a new task [22,25]. In this study, our goal was to provide an objective US diagnostic method by utilizing both a CNN trained from scratch and TL with fine-tuning and gradual unfreezing to differentiate between malignant and benign SGTs. We aimed to assess the diagnostic potential of ultrasound imaging alone when encountering a newly diagnosed SGT. We included all types of tumors found within the salivary gland, including metastatic carcinoma and lymphoma. Factors such as the stage of cancer, a patient’s cancer history, or the presence of adjacent lymphadenopathy or distant metastasis were not considered during our analysis. Additionally, we employed gradient-weighted class activation mapping (Grad-CAM) to visualize the regions that the model focuses on [26]. Grad-CAM uses the gradients with respect to the feature maps of the last convolutional layer to generate a map highlighting the regions that the model focuses on. This can provide a better understanding of what the model has learned.

## 2. Materials and Methods

### 2.1. Ethical Considerations

This study was performed in accordance with the Declaration of Helsinki and obtained approval from the institutional ethical review board (IRB No. 111199-E and No. 112136-E). Informed consent was waived due to retrospective and anonymous study design. The study did not impact the patients’ treatment or outcome.

### 2.2. Inclusion Criteria

**Figure 2:**
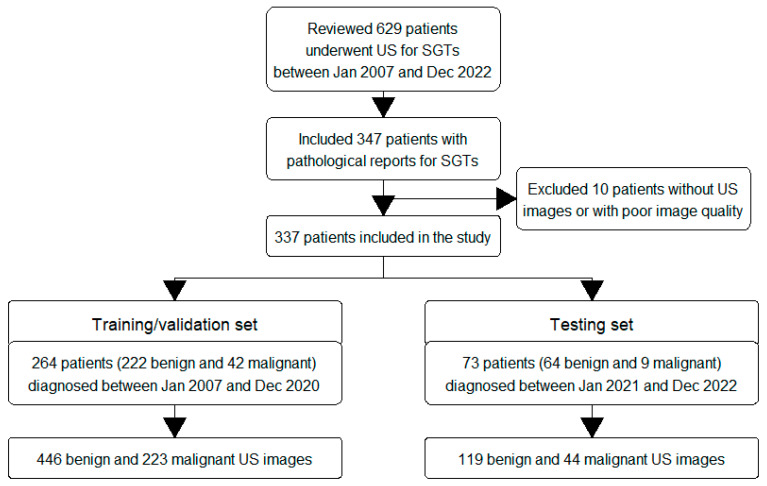
Flow chart to illustrate the study’s inclusion and exclusion criteria. Abbreviation: US, ultrasound; SGT, salivary gland tumor.

This retrospective study was conducted at a tertiary medical center. We reviewed patients who visited our outpatient department between January 2007 and December 2022 and underwent US examinations for suspected major salivary gland tumors. The US examinations were performed by experienced otolaryngologists. We included 337 adult patients, aged 20 years or older, who underwent further operations or core needle biopsies (CNB) and for whom pathological reports were obtained. CNB was performed when patients were deemed unsuitable for open surgery. Pathological diagnoses based on pathological reports were used as the ground truth for classifying tumors as malignant or benign. Patients without US images or with poor image quality were excluded. The flow chart of the inclusion and exclusion criteria is shown in Figure 2.

### 2.3. Data Collection

To effectively build and evaluate the model, we divided the patients into two sets. The training/validation set, which consisted of 264 patients diagnosed between January 2007 and December 2020, was used to establish and validate the model. The testing set, which included 73 patients diagnosed between January 2021 and December 2022, was used to assess the model’s ability to make predictions. We collected demographic data (age and sex), tumor characteristics (side, location, and size), and pathological reports for the included patients (Supplementary Table S1). US examinations were performed using a Toshiba Aplio 500 (Canon Medical Systems, Tochigi-ken, Japan) with a 5–14 MHz linear-array transducer in B-mode. The US images were retrieved from the picture archiving and communication system (PACS). The training/validation set comprised 222 benign and 42 malignant SGTs, and the testing set comprised 64 benign and 9 malignant SGTs. To address the data imbalance, we collected a larger number of US images for malignant SGTs and a smaller number for benign SGTs. The collected US images encompassed different views of the tumors, including the long or short axis and horizontal or vertical view, along with the neighboring regions. As a result, we collected a total of 446 benign and 223 malignant US images in the training/validation set and 119 benign and 44 malignant US images in the testing set (Figure 2).

### 2.4. Data Preparation

**Figure 3:**
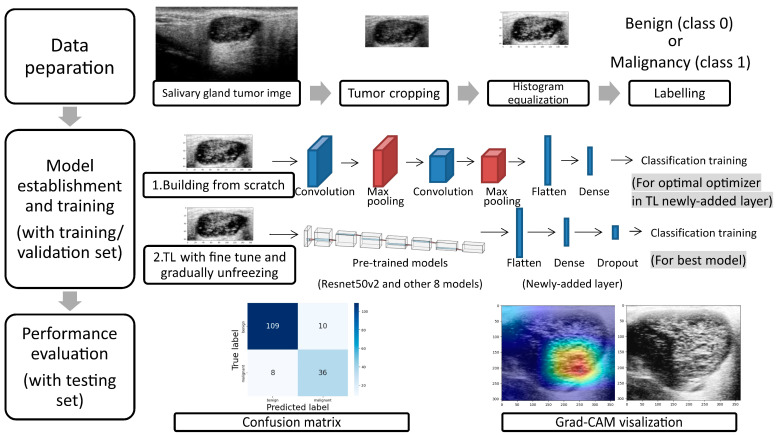
The study protocol. Note: In the first section of our model establishment process, we constructed a prediction model from scratch. This was carried out to identify the optimal optimizer for the newly added layer in the subsequent section. In the second section, we utilized transfer learning, incorporating fine-tuning and gradual unfreezing techniques. We selected nine pre-trained models and added new layers to them. The model that demonstrated the highest validation accuracy was chosen for further evaluation of its diagnostic performance on the testing set. Abbreviation: Mal, malignant; TL, transfer learning.

The study protocol is presented in Figure 3. First, to reduce the noise signal in the US images and focus specifically on the tumor, the images were cropped to encompass the entire tumor and its surrounding region in a rectangular region. Second, to address variations in brightness settings among different otolaryngologists, histogram equalization was applied to all cropped US images. The resulting images were labeled either as benign or malignant according to the pathological diagnosis for the subsequent experiments, with class 0 indicating benignity and class 1 indicating malignancy.

### 2.5. Model Establishment

We trained our model using the Python framework on Google Colaboratory (Colab) with an NVIDIA T4 GPU (NVIDIA Corp., Santa Clara, CA, USA). Colab provides free GPU resources and serves as an online Jupyter Notebook. The input image was resized to 150 × 150 grayscale for the following experiments. The optimal model was determined based on the results of the validation set. We employed binary cross-entropy as the loss function, which is defined as follows:

$$L_{\mathrm{BCE}}=-\frac{1}{N}\overset{N}{\underset{i=1}{\sum }}((Y_{I}*\log\left(P_{I}\right)+(1-Y_{I})*\log\left(1-P_{I}\right))$$

where *N* represents the total sample size. For a random sample, *Y_I_* represents its truth label, *P_I_* represents its prediction probability of class 1, and (1 – *P_I_*) represents its prediction probability of class 0.

In the first section, we built the prediction model from scratch. For this experiment, we randomly split the training/validation set into 20% for training and 80% for validation. A CNN model was constructed from two alternating convolution layers and max pooling layers, followed by a classification layer. The detailed information of the model and the number of neurons is as follows: convolution (16), max-pooling (2 × 2), convolution (32), max-pooling (2 × 2), flatten, dense (512), and classification. We examined different optimizers, the number of convolution layers, kernel sizes, the presence of the dropout layer, dropout percentages, and the presence of batch normalization. The optimizer options included SGD, RMSprop, Adagrad, Adadelta, Adam, Adamax, and Nadam. The convolution layers ranged from two to six layers. The kernel size options were 3 × 3, 5 × 5, and 7 × 7. The dropout layer was tested with percentages of 10%, 30%, and 50%. The batch size was set to 16, and the epoch was set to 30 for training. The goal of this experiment was to determine the most suitable optimizer for classifying SGTs in the newly added layer of the second section and evaluate the diagnostic performance of these handcrafted models.

In the second section, we employed TL with fine-tuning and gradual unfreezing using a pre-trained model. The input grayscale image was converted to the RGB channel by replicating grayscale image pixels. The pretrained model used in this study included those that were ever applied in the classification of liver US images (ResNet50V2, MobileNetV2, EfficientNetB0, DenseNet121, NASNetMobile, and InceptionResNetV2) [27,28,29], breast US images (Xception and InceptionV3) [30], and thyroid US images (VGG16 and InceptionV3) [31,32]. We removed the top layer of these pretrained models and connected them to a new dense layer (512 neurons), a dropout layer (20%), and a classification layer. Due to the limited dataset size, we applied 5-fold cross-validation to select models with superior performance. The batch size was set to 16, and the training epoch was set to 40. Three models with higher accuracy were chosen for fine-tuning and gradual unfreezing. During the fine-tuning process with gradual unfreezing, we unfroze the layers of the pretrained model from top to bottom in a step-by-step manner. Initially, all layers of the pretrained model were frozen, and only the dense and classification layers were trained. Subsequently, the last block of the pretrained model was unfrozen and retrained. We continued unfreezing more layers and evaluated the 5-fold cross-validation results at each stage. If no further improvement in the 5-fold average validation accuracy was observed, we stopped training and utilized the previous training parameters for further evaluation. The batch size was set to 16, and the training epoch was set to 20. Finally, we selected the model with the highest validation accuracy to assess its diagnostic performance on the testing set. To visualize the model’s predictions, we utilized Grad-CAM.

### 2.6. Statistical Analysis

All statistical analyses were performed using STATA software, version 14.0 (Stata Corporation, College Station, TX, USA). The clinical characteristics are presented as the mean and standard deviation (SD) or number and percentage (%). Categorical data were compared using the chi-square test, while continuous data were compared using the t-test. A confusion matrix with accuracy, sensitivity, specificity, positive predictive value (PPV), and negative predictive value (NPV) was obtained by applying the model with the highest validation accuracy to the testing set. A p-value less than 0.05 was considered statistically significant in this study.

## 3. Results

The flow chart of the inclusion and exclusion criteria is presented in Figure 2. We included 264 patients in the training/validation set and 73 patients in the testing set. The clinical characteristics are summarized in Table 1. There were no significant differences in age, sex, tumor side, tumor location, or tumor size between the training/validation set and the testing set (all *p* values > 0.05). Among these patients, 286 had benign tumors, and 51 had malignant SGTs (Table 2). The most common benign tumors were pleomorphic adenoma (40%) and Warthin’s tumor (37%), while poorly differentiated or undifferentiated carcinoma (26%) was the most common malignant tumor.

For the subsequent experiments, we collected a total of 446 benign and 223 malignant US images of SGTs in the training/validation set and 119 benign and 44 malignant US images of SGTs in the testing set. First, we constructed the CNN model from scratch (Table 3). The results indicated that the Adam optimizer achieved higher training and validation accuracy than the other optimizers. However, other methods, such as increasing layers, changing kernel size, or incorporating dropout or batch normalization, did not significantly improve the validation accuracy.

**Figure 4:**
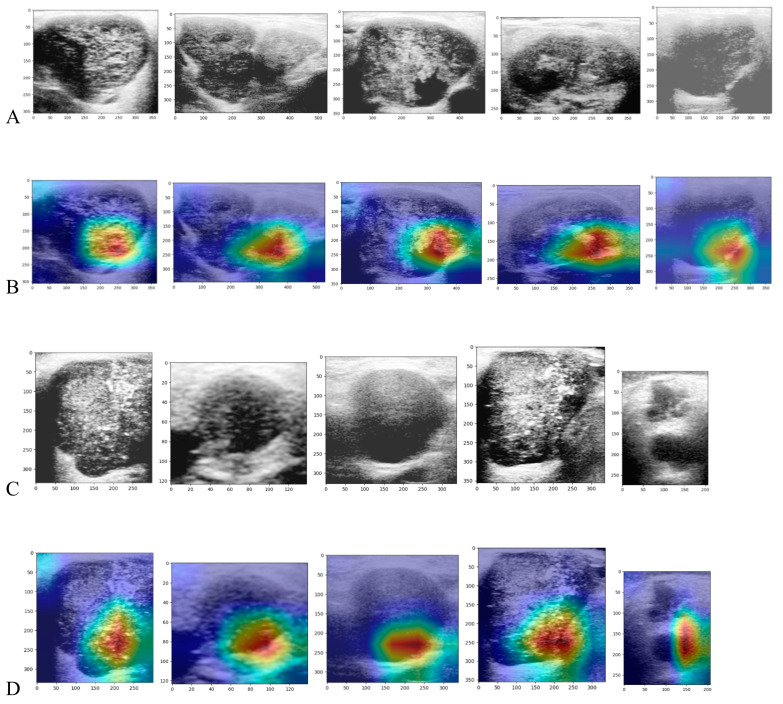
The example of ultrasound images from the testing set and its corresponding Grad-CAM image. Note: The first line displays US images of benign SGTs (**A**), while the second line shows their Grad-CAM images (**B**). The third line depicts US images of malignant SGTs (**C**), and the fourth line presents their Grad-CAM images (**D**). Abbreviation: Grad-CAM, gradient-weighted class activation mapping; US, ultrasound; SGT, salivary gland tumor.

Second, due to the limited dataset, we applied TL with 5-fold cross-validation (Table 4). Among the nine pretrained models evaluated in this study, DenseNet121, VGG16, and ResNet50V2 demonstrated higher average validation accuracies (0.798, 0.789, and 0.771, respectively) during 5-fold cross-validation. Therefore, we selected these three models for further fine-tuning and gradual unfreezing under 5-fold cross-validation (Table 5). The results indicated that ResNet50V2 and DenseNet121 had similar average validation accuracies (0.920 vs. 0.919). However, in the testing set, ResNet50V2 exhibited higher accuracy than DenseNet121 (0.890 vs. 0.753). By utilizing the pretrained ResNet50V2 model with fine-tuning and gradual unfreezing, the diagnostic performance on the testing set achieved an accuracy of 89.0%, a sensitivity of 81.8%, a specificity of 91.6%, a PPV of 78.3%, and an NPV of 93.2% (Table 6). Grad-CAM was employed to visualize the model’s outputs. Figure 4 illustrates the Grad-CAM in the testing set, showing the important regions that the model used to classify the SGT.

## 4. Discussion

This study compared the diagnostic performance of the CNN trained from scratch and TL with fine-tuning and gradually unfreezing for differentiating between malignant and benign SGTs based on US images. A separate testing set, collected between January 2021 and December 2022, was used to evaluate the model’s performance. The results demonstrated that TL with fine-tuning and gradual unfreezing outperformed the CNN trained from scratch (Table 3, Table 4 and Table 5). Specifically, the ResNet50V2 model with fine-tuning and gradual unfreezing achieved the highest diagnostic accuracy compared to DenseNet121 and VGG16 (Table 4). In the testing set, the model demonstrated much higher sensitivity (81.8%) and accuracy (89.0%) with similar specificity (91.6%) compared to the subjective US features reported in a previous meta-analysis, which had a pooled sensitivity of 62.9% and specificity of 92.0% [7]. These findings indicate that our model is an effective and objective diagnostic method for accurately classifying SGTs using US images and may offer better diagnostic performance than subjective US features.

The presence of various benign and malignant tumor types (Table 2), as well as the varying degrees of malignancy, poses a challenge for diagnosing SGTs based on US images alone [5]. Traditional subjective US features used to identify malignant SGTs include vague boundaries, irregular shapes, the presence of calcification, the presence of lymphadenopathy, and the absence of posterior acoustic enhancement [11,13]. In our previous study, we evaluated these subjective US features for differentiating between malignant and benign SGTs, resulting in a sensitivity of 58%, specificity of 89%, and accuracy of 85% [13]. We also assessed US elastography, including shear wave elastography and strain elastography, but observed poor diagnostic performance. In our current study, the ResNet50V2 model with fine-tuning and gradual unfreezing demonstrated higher sensitivity (81.8%), specificity (91.6%), and accuracy (89.0%) compared to our previous study, which relied on subjective US features [13]. Moreover, this performance was comparable to that of CT (pooled sensitivity of 83.0% and specificity of 85.1%) and MRI (pooled sensitivity of 80.7% and specificity of 88.6%), as reported in previous meta-analyses [7]. Our model demonstrated the potential to classify SGTs more effectively than subjective US features, with a diagnostic performance similar to that of CT or MRI. By using the feature maps learned by kernel filters, our model offers a distinct perspective from the subjective US features in diagnosing SGTs, potentially leading to a more reliable diagnostic outcome.

TL has been gradually used in US image classification for organs, such as the thyroid [33], breast [34], and liver [35], but it is less commonly used for SGT. Wang et al. reported the first study on the application of TL in diagnosing SGTs using US images [20]. They compared different TL models and reported diagnostic accuracies of 79% for ResNet50, 77% for DenseNet121, and 80% for EfficientNetB3. In our study, we collected as many pretrained models as possible to select those with the highest applicability to our dataset. We then used fine-tuning and gradual unfreezing to further train these models. Fine-tuning trains selected layers of the pretrained model along with the newly added top layers during the training process. Combined with gradual unfreezing, the model can gradually train newly unfrozen layers to achieve higher diagnostic performance. Another method employed in our study is K-fold cross-validation, which is commonly used in DL for medical image classification, including thyroid US images [36,37]. K-fold cross-validation helps mitigate selection bias in small sample sizes by dividing the data into k subsets and iteratively using one subset as the validation data while the remaining subsets serve as the training data. This approach allows for obtaining an average performance of the model and reduces overfitting to a specific subset of the data. We evaluated nine pretrained models using TL with 5-fold cross-validation and selected three models with higher average validation accuracy (78.9% for VGG16, 77.1% for ResNet50V2, and 79.8% for DenseNet121; Table 4). After fine-tuning and gradual unfreezing, the 5-fold average validation accuracy improved to 92.0% for ResNet50V2 and 91.9% for DenseNet121 (Table 5). This result confirmed the additional effect of fine-tuning and gradual unfreezing on TL. By implementing fine-tuning with gradual unfreezing and K-fold validation, our model achieved a better diagnostic performance in the testing set, with an accuracy of 89.0%, a sensitivity of 81.8%, a specificity of 91.6%, a PPV of 78.3%, and an NPV of 93.2%. These results outperformed those of the previous study [20].

Furthermore, for the enhanced interpretability of our model, we employed grad-CAM to visualize the decision-making process. Grad-CAM identifies the regions that the model focuses on by utilizing the gradients between the output and the last convolutional layer. These gradients are multiplied by their corresponding feature maps and combined to generate a heatmap. By overlaying the heatmap on the original image, a Grad-CAM image is created. Grad-CAM highlights the area that contributes the most to the predicted class, facilitating a better understanding of the model’s prediction. As shown in Figure 4, the region that our model focused on was mostly located in the middle and right lower portion of the tumor. Our model demonstrated its ability to objectively classify SGTs, which could assist in preoperative evaluation. If the tumor is determined to be benign, then extracapsular dissection or superficial parotidectomy may be adequate. However, if the tumor is determined to be malignant, then wide excision with lymph node dissection may be necessary.

## 5. Limitations

There were several limitations to this study. First, this study was a retrospective study and not a randomized controlled trial, which may have introduced selection bias and limited the generalizability of our findings. Second, the sample size was relatively small, consisting of 337 patients with 832 US images of SGTs. While we employed K-fold cross-validation to mitigate the impact of the small sample size, the model developed based on this dataset may not accurately represent other populations. Third, we obtained a larger number of ultrasound images from patients with malignant tumors than from those with benign tumors. This discrepancy may introduce selection bias into our study. Fourth, despite the application of histogram equalization, we could not entirely eliminate the potential effects of operator variability. Furthermore, variations in US machines across different hospitals may also influence the model’s predictions. Therefore, it is crucial to conduct further prospective studies and involve multiple facilities to effectively validate and apply this model.

## 6. Conclusions

In this study, we evaluated CNN models trained from scratch and TL with various pretrained models using the online platform Colab. Our findings demonstrated that the pretrained ResNet50V2 model with fine-tuning and gradual unfreezing exhibited superior diagnostic accuracy compared to other models. Additionally, we employed Grad-CAM to elucidate the underlying reasons behind the model’s diagnoses. Our study provides an effective and objective US method for distinguishing between malignant and benign SGTs.

## Figures and Tables

**Table 1:** Comparing the clinical characteristics between the training/validation and testing sets.

Demographic Data (Mean (SD) or N (%))	Training/Validation	Testing	*p* Value
	N = 264	N = 73	
Age, year	53 (14)	54 (15)	0.493
Sex			0.453
Female	110 (42%)	34 (47%)	
Male	154 (58%)	39 (53%)	
Side			0.837
Right	141 (53%)	38 (52%)	
Left	123 (47%)	35 (48%)	
Location			0.302
Parotid gland	206 (78%)	61 (84%)	
Submandibular gland	58 (22%)	12 (16%)	
Tumor size			
Short axis, cm	1.7 (0.6)	1.6 (0.6)	0.336
Long axis, cm	2.5 (1.0)	2.4 (0.9)	0.461
Short–long-axis ratio	0.7 (0.2)	0.7 (0.1)	0.586
Pathological diagnoses			0.450
Benign tumors	222 (84%)	64 (88%)	
Malignant tumors	42 (16%)	9 (12%)	

**Table 2:** Pathological reports for the patients included in the study.

Pathological Reports	All	Training/Validation	Testing
	N = 337	N = 264	N = 73
Benign salivary gland tumors	286	222	64
Pleomorphic adenoma	114 (40%)	91 (41%)	23 (36%)
Warthin’s tumor	106 (37%)	83 (37%)	23 (36%)
Other benign tumors (basal cell adenoma, oncocytoma, hemangioma, chronic sialadenitis, IgG4-associated sialadenitis, etc.)	66 (23%)	48 (22%)	18 (28%)
Malignant salivary gland tumors	51	42	9
Poorly differentiated/undifferentiated carcinoma	13 (26%)	12 (29%)	1 (11%)
Mucoepidermoid carcinoma	12 (24%)	7 (17%)	5 (56%)
Metastatic carcinoma	10 (20%)	9 (22%)	1 (11%)
Lymphoma	5 (10%)	5 (12%)	0 (0%)
Lymphoepithelial carcinoma	4 (8%)	3 (7%)	1 (11%)
Adenoid cystic carcinoma	2 (4%)	2 (5%)	0 (0%)
Adenocarcinoma,	2 (4%)	1 (2%)	1 (11%)
Acinic cell carcinoma	1 (2%)	1 (2%)	0 (0%)
Carcinoma ex pleomorphic adenoma	1 (2%)	1 (2%)	0 (0%)
Salivary duct carcinoma	1 (2%)	1 (2%)	0 (0%)

**Table 3:** Constructing CNNs from scratch and evaluating their performance.

Optimizer	SGD	RMSprop	Adagrad	Adadelta	Adam	Adamax	Nadam
ACC	0.71	0.93	0.70	0.67	0.99	0.86	0.99
LOSS	0.56	0.21	0.59	0.62	0.04	0.33	0.05
VAL_ACC	0.61	0.62	0.67	0.67	0.68 *	0.68	0.63
VAL_LOSS	0.70	1.01	0.67	0.63	1.54	0.69	1.49
**Layer**	2	3	4	5			
ACC	0.99	0.97	0.79	0.63			
LOSS	0.04	0.09	0.47	0.61			
VAL_ACC	0.68 *	0.60	0.72	0.67			
VAL_LOSS	1.54	1.71	0.85	0.63			
**Kernel size**	3 × 3	5 × 5	7 × 7				
ACC	0.99	0.98	0.97				
LOSS	0.04	0.07	0.07				
VAL_ACC	0.68 *	0.63	0.51				
VAL_LOSS	1.54	1.86	1.89				
**Dropout**	No	10%	30%	50%			
ACC	0.99	0.92	0.83	0.78			
VAL_ACC	0.68 *	0.59	0.66	0.68			
**Batch normalization**	No	Yes	+dropout 10%	+dropout 50%			
ACC	0.99	0.90	0.81	0.73			
VAL_ACC	0.68 *	0.68	0.51	0.56			

Note. * Variables with the highest diagnostic performance (higher accuracy and lower loss) in each experiment. Abbreviation: ACC: accuracy; VAL_ACC: validation accuracy; VAL_LOSS: validation loss.

**Table 4:** Transfer learning with feature extraction and 5-fold cross-validation is employed to select models with superior diagnostic performance.

Model	VGG16	ResNet50V2	MobileNetV2	EfficientNetB0	DenseNet121	Xception	NASNetMobile	InceptionV3	InceptionResNetV2
AVG_ACC	0.999	0.999	0.540	0.473	0.996	0.992	0.997	0.998	0.995
AVG_LOSS	0.030	0.007	0.924	0.924	0.020	0.032	0.018	0.019	0.033
AVG_VAL_ACC	0.789 *	0.771 *	0.505	0.693	0.798 *	0.767	0.741	0.737	0.756
AVG_VAL_LOSS	0.633	1.348	0.694	0.441	0.720	1.022	0.981	0.996	0.967

Note. * Models with higher 5-fold average validation accuracy. Data of each fold were included in Supplementary Table S2. Abbreviation: AVG_ACC: average accuracy; AVG_LOSS: average loss; AVG_VAL_ACC: average validation accuracy; AVG_VAL_LOSS: average validation loss.

**Table 5:** Transfer learning with fine-tuning and gradual unfreezing, combined with 5-fold cross-validation, is employed to enhance the diagnostic performance of selected models.

Model	DenseNet121	VGG16	ResNet50V2
Unfreeze layer	conv4_block13_0_bn	Block4_conv1	conv4_block5_preact_bn
Learning rate	0.00001	0.0001	0.0001
AVG_ACC	1.000	0.732	0.996
AVG_LOSS	0.005	0.376	0.021
AVG_VAL_ACC	0.919	0.618	0.920 *
AVG_VAL_LOSS	0.237	0.998	0.566
TEST_ACC	0.753	0.667	0.890
TEST_LOSS	0.543	0.692	0.527

Note. * Models with the highest 5-fold average validation accuracy. Data of each fine-tuning were included in the Supplementary Table S3. Abbreviation: AVG_ACC: average accuracy; AVG_LOSS: average loss; AVG_VAL_ACC: average validation accuracy; AVG_VAL_LOSS: average validation loss; TEST_ACC: testing accuracy; TEST_LOSS: testing loss.

**Table 6:** The diagnostic results and performance of ResNet50V2 with fine-tuning and gradual unfreezing in the testing set.

ResNet50V2 with Fine-Tuning and Gradual Unfreezing	Testing Set
Diagnostic result	
True positive	36
False negative	8
False positive	10
True negative	109
Diagnostic performance	
Accuracy	89.0%
Sensitivity	81.8%
Specificity	91.6%
Positive predictive value, PPV	78.3%
Negative predictive value, NPV	93.2%

## Data Availability

The data presented in this study are available in the Supplementary Materials.

## Supplementary Materials

The following supporting information can be downloaded at: https://www.mdpi.com/article/10.3390/diagnostics13213333/s1, Supplementary Table S1: Clinical characteristics of included patients; Supplementary Table S2: Data of each fold in Table 4; Supplementary Table S3: Data of each fine-tuning in Table 5.
